# Pathology, Practical Medicine, and Therapeutics

**Published:** 1838-01

**Authors:** 


### PATHOLOGY, PRACTICAL MEDICINE, AND THERAPEUTICS.

### DIFFERENT VIEWS OF THE NATURE AND TREATMENT OF TETANUS.

I. *A Case of Tetanus, confirmatory of the Views of* Bellingeri. By Signor Morotti Francesco, of Vercelli.

[We give this case as we find it in the original Journal, with the observations of the author. We will only remark that, whether the case is confirmatory or not of the views of the celebrated Italian physiologist, the dependence of particular symptoms observed during life on particular organic changes observed after death, is by no means logically proven. The case is nevertheless interesting, as establishing the locality of the disease viewed as a whole.]

Dominico Pozzo, æt. twenty-three, of a strong constitution, after working hard and perspiring profusely in the hayfield, was seized in the evening of August 19 with rigors and cold sweats. The next day he had fever, with violent pain in the forehead and vertex, drawing the head downwards and forwards, and stitches in the side during inspiration; and on the day following, a pain down his neck in addition to the other symptoms. On the 27th, notwithstanding a general bleeding and leeches to the throat, the symptoms increased, and the patient perceived a drawing of the whole trunk forwards. The head was bent to the chest, the facial muscles were contracted, the lower extremities were spasmodically bent and drawn towards each other, and the motions of abduction and elevation of the upper arms were painful and difficult, but the forearms and hands moved easily in every direction. Received in this state into the hospital at Vercelli, he was bled largely. The blood was rather buffed. The next day, 28th, the emprosthotonos was found to be changed to episthotonos; head and neck drawn back and a little to the left side, pain in the occiput, which by degrees became more piercing, that in the head having ceased; sight, hearing, and intellect unimpaired, speech hesitating and slow, features drawn and convulsed into a risus sardonicus, lower extremities spasmodically extended, upper arms slightly extended and abducted, respiration difficult, with abundant expectoration, pulse slow and contracted. An inflammation of the cerebellum being suspected, he was treated accordingly.

31st. Trunk curved backwards, with acute pain and severe spasms of the head and neck, alæ nasi drawn upwards, lips drawn apart, lower jaw almost closed with trismus, pulse hard and very frequent.

*September* 1. Opisthotonos, pain in occiput and neck less, but extending all down the spine, heat of skin natural, head and chest bathed in sweat, pulse variable. In the evening, stupor, opisthotonos, sensation impaired, legs widely extended, but allowing flexion till a fresh spasm extended them. Death at midnight.

*Examination thirty-two hours after Death.* No stiffness nor curvature of neck or trunk. Pia mater and arachnoid generally injected, some serum in the cavity of the arachnoid, and a little effused blood between the convolutions of both hemispheres. Substance of the brain hard and resistant to the scalpel, and somewhat engorged, a little serum in left ventricle. State of cerebellum was the same. The membranes of the spinal cord were injected, and the posterior columns of the cord were soft as pap, and covered with a puriform serum; the anterior columns presented evident marks of inflammation, and their medullary matter was as hard as that of the cerebral lobes. The left pleura was inflamed and adherent, and the right lung, hepatized in various parts, floated in a large quantity of extravasated blood.

In this case it is evident that the inflammation began in the cerebral lobes and their membranes, as was indicated by the pain in the head, which, setting in on the first day, extended at length to the neck, and probably to the anterior columns only of the cord. To this inflammation, continued till the eighth day, must be attributed the emprosthotonos which appeared on that day, and the state of adduction of the extremities; thus confirming the opinion of Bellingeri that the cerebral hemispheres preside over the motions of flexion and adduction, and that when they, or the anterior columns of the spinal cord, are irritated, emprosthotonos is the result.

On the ninth day the disease attacked the cerebellum, as was proved by the occipital pains, which began on that day, and by the change from emprosthotonos to opisthotonos, with convulsive movements of extension and abduction of the extremities. The backward curvature of the trunk on the eleventh day appears to be owing to the supervening inflammation of the posterior columns of the cord as revealed by the dissection; and the general opisthotonos to the affection of the cerebellum and cord together. This again confirms the opinion of Bellingeri that the cerebellum and posterior columns determine the movements of extension and abduction; and that their irritation produces opisthotonos.

*Annali di Medicina. Gennajo,* 1835.

II. *Case of Tetanus, with Remarks.* By Dr. BRUGGEMANN, of Magdeburg.

ON entering the hospital one morning, Dr. Bruggemann remarked a young man pacing the ward, apparently wrapped in meditation, with his face directed towards the ceiling, but with something comical in his expression; which, together with his inattention to surrounding objects, led Dr. B. at first to consider him as insane. On questioning him, however, it appeared that, on the 8th of November, he had experienced some difficulty in deglutition, with slight pains in the neck; on the 9th, he complained of difficulty in masticating, and could not open his mouth fully; on the 10th, there was stiffness of the neck; and, on the 11th, (the day in question,) the free motion of the limbs was impeded, and he felt uncomfortable whilst lying, sitting, or even standing, and found himself most at ease when walking. The face was flushed, the pupils enlarged, and the mouth capable of being opened only about half an inch. The masseteric muscles felt hard; deglutition was difficult; he suffered from thirst and hunger. The arms were fixed in a semiflexed position; he could not bend the body forwards; and the dorsal muscles felt hard to the touch. The respiration was free; pulse 110, small, and soft; temperature of skin natural. Every few seconds he suffered from violent shooting pains, which passed like an electric shock from the spine over the chest, and were accompanied by a convulsive twitching of the muscles, particularly those of the back, neck, and face.

Twelve cupping-glasses were applied along the spine, and forty leeches to the nape of the neck; six ounces of blood were drawn from the arm, and two grains of calomel given every two hours. During the night, the shooting pains were more severe; the patient had profuse acid perspiration, but the other symptoms were mitigated. On the 12th, he had an alkaline bath; opium was added to the calomel, and a blister was applied to the nape of the neck. On the 13th, the pains were somewhat relieved, but the other symptoms continued much the same. The bath was repeated, and two grains of opium were given every two hours, alternately with a decoction of bark containing muriatic acid. The patient complained of hunger and thirst, the respiration was anxious and accelerated, and the pulse at the wrist could not be counted; the skin was moist, and the patient was affected with giddiness. At six, there was some delirium, which was followed, at nine, by extreme anxiety; the limbs were then powerless and relaxed, the respiration slow, pulse 160; the sphincters paralyzed. Death followed in about an hour.

On opening the spinal column, about thirty-six hours after death, some dark-coloured blood escaped, and an extravasation was found between the second and third cervical vertebræ. A second extravasation extended from the seventh cervical to the first lumbar vertebra, and the whole spinal canal contained a large quantity of dark half-coagulated blood. The vessels of the spinal dura mater were every where injected, and the vasa spinalia were gorged with blood. The substance of the spinal marrow was normal. The superficial cerebral vessels were gorged with blood, but the large vascular branches in the base of the cranium were empty.

After detailing this case, Dr. Bruggemann proceeds to theorize on the cause of tetanus; and, dismissing inflammation as unsatisfactory, seems rather inclined to acknowledge rheumatism as one of its most frequent causes. As the results of his own experience, he would recommend the following mode of treatment, founded on the conviction that the disease *must* be curable, could we only have sufficient boldness to stand firm and carry through some energetic plan of treatment. In the first place, he would commence giving opium in doses of three or four grains, repeated every quarter of an hour till sleep was induced, which is the great point to be gained. On the patient awakening, the most powerful stimulants and tonics, such as wine and quinine, are to be administered; to be followed, should the convulsions still continue, with repeated doses of opium till sleep again ensues; to be again succeeded by stimulants and tonics.

*Wochenschrift für die gesammte Heilkunde.* Nos. 19, 20, and 21. 1837.

### III. *Tetanus caused by exposure to Wet, and cured by a Moral Impression (?)* By Dr. Ferrus.

Dr. Ferrus relates a case of a young man, who, having danced a whole evening in company with his sweetheart, and having drunk much, lay down on the turf, and slept during a storm, to which he was completely exposed. On awaking, he felt much pain in his limbs, and had a good deal of fever during the night. The next morning, trismus came on, and was followed by other tetanic symptoms. These soon became so severe as to prevent his lying down in bed; a convulsion of the whole body occurred nearly every minute; there was complete inability to swallow and to void urine; total absence of sleep. The treatment consisted in bleeding from the arm, cupping and the application of the actual cautery along the spine; opium was given in large doses, by means of a canula inserted into the mouth. These remedies produced no effect; the patient continued to get worse, and on the seventh day the physician left him in despair to the care of the priest and the notary. On leaving the house, he met the young woman to whom his patient was attached, and who was in the utmost distress at being prevented from visiting him by the interference of his mother, who was opposed to their union. By the assistance of the kind priest he succeeded in procuring an interview between the lovers, which was followed by the happiest effects. Abundant tears were shed; the convulsions diminished, so that in the evening he could be placed in bed, when he obtained three hours' sleep; there was an abundant secretion of urine, but the bowels remained obstinately constipated for a considerable time. The patient now continued to recover gradually, and was finally restored to health.

*Journal des Connaissances Medico-Chirurgicales.* *May*, 1837.

IV. *Of the Successful Employment of Colchicum in Tetanus, in Hayti.* By WILLIAM G. SMITH, M.D., of Port au Prince.

IN Hayti, as in all warm climates, tetanus prevails to a considerable extent, but it would seem most confined to the natives, as I have never known a case to occur in an European, or any stranger whatever. It also appears, that persons in this country, from the age of five years to thirty, or thirty-five, are most predisposed to attacks of this disease. In the course of little more than two years and a half, nine cases of tetanus have fallen under my observation, and the greater number of these were under my own care. Women do not seem to be so susceptible of tetanus in any of its varieties as men: of the nine cases, only one having occurred in a female. Of the nine cases, two were traumatic, and were induced by very slight wounds, the remaining seven were idiopathic, no direct cause being present.

| | |
|---|---|
| Traumatic Trismus . . . | 2 males, under the age of 20, 1 died, 1 cured. |
| Idiopathic Opisthotonos | 3 males, under the age of 30, 1 died, 2 cured. |
| | 1 female, under the age of 40, died. |
| | 2 males, under the age of 25, cured. |
| Idiopathic Pleurosthotonos . | 1 male, aged 15, died. |

Total, 9: cured, 5; deaths, 4.

The last four cases of tetanus which have come under my care, were treated with the Colchicum Autumnale, and three out of the four recovered. The following is the general course I pursue, in the treatment of the disease: on being called to a case of tetanus my attention is first turned to the actual state of the bowels; constipation in this disease is at least a grave symptom, and should be relieved by injections. I prefer some emollient fluid for this purpose; because I believe that in almost all cases, there is, conjoined with costiveness, some degree of intestinal irritation. I commenced by administering an enema of lbj. decoction of either flaxseed or the okra, (Ketmia gombo,) cum. ol. ricini. ℥iij. or ℥iv. for an adult. After this, fifty or sixty leeches are applied to the spine, from the neck to the sacrum; in my particular practice, I have made choice of the scarificators and cupping-glasses, and the result has been most satisfactory. When the muscles of the jaws and neck are affected, as is most usually the case, leeches are also applied to the mastoid processes; the moment the cups or leeches, whichever may have been used, are removed, cloths wrung out of a strong solution of the muriate of ammonia are constantly applied to the whole of the vertebral column, the back and neck. Internally I administer the vinous tinct. of colchicum, commencing with ʒss.; the dose is increased every half-hour, and repeated until emesis or catharsis has been produced. As soon as one or other of these effects is obtained, this remedy is to be discontinued; if there afterwards occur any colic or griping, with exhaustion, as in all probability there will, I am in the habit of giving the spt. mindereri ℥ss. every hour, with the addition of one-fourth of a grain of acetate of morphine, in solution. If the surface be cold, and there be symptoms of collapse, warm applications are made to the extremities and the axilla; and the muriated solution is discontinued. The seeds of the colchicum appear more permanent in effect than the bulb. I accordingly make a tincture as follows:—℞. Sem. colch. sicc. ℥ij.; Vin. albi, hisp. lbj. Infund. &c.

All the cases of tetanus which have fallen under my care have been, with two exceptions, treated as above stated, with a very happy result. The exceptions were the first two cases I treated in this island, and in which I employed opium, general venesection, terebinth, &c.; the patients were put in a warm bath, but on being taken out of the bath, the spasms became more alarming, the limbs more rigid, and in one instance, death immediately followed. I shall never again treat tetanus, while in the West Indies, as recommended in the books. I was first induced to employ the colchicum for the cure of tetanus from the accounts given by Mr. Haden, of London, of its good effects in chronic rheumatism, and inflammatory diseases, as well as from previous experience, while resident Physician at the House of Refuge in New York. Dr. Ives, of that city, frequently employed the tincture in rheumatism. The effect I invariably observed it to produce led me to believe that the colchicum possessed qualities both as diaphoretic, diuretic, and actively cathartic, besides being antispasmodic and anodyne. I am now certain that in tetanus, it acts in a manner altogether peculiar. In tetanus there is obstinate constipation, violent spasms, muscular rigidity, with pain, often retention or suppression of urine. Now the colchicum, when genuine, appears to combine in itself sufficient qualities to control several of these symptoms at once. It, however, does not invariably promote vomiting; this may depend on its genuineness, but it almost invariably acts upon the kidneys, the skin, and the bowels.

*The Jamaica Physical Journal, for June,* 1836.

V. *Tetanus cured by Acetate of Morphine, applied endermically.*
By Dr. Henry.

This was the case of a young man, over whom passed the wheel of a carriage, by which two ribs and several of the metacarpal bones of the right hand were fractured, with great laceration of the soft parts. He was bled, and the fractured parts replaced. Until the fifteenth day, all went on favorably, when there suddenly occurred the symptoms of tetanus; fixed look, stupor, trismus, contraction of the muscles of the neck and superior part of the thorax, &c. A grain and a half of acetate of morphine was spread upon the surface of the wound, and an ounce of syrup of morphine administered internally, by means of a tube passed through the interval left by a tooth which had been extracted. The symptoms ceased during the following day, when the same remedies were employed; the next day, they were omitted, when the symptoms returned more formidably than before; the patient sitting upon his bed with his head almost between his legs, and being unable to lift it, (Emprosthotonos.) An abraded surface was made by ammonia, and two grains of acetate of morphine were applied to it; the application was continued, and the patient recovered.

*Bulletin de l'Academie Royale de Médecine.* No. 2. 1836.

---

*On the present Predominant Views regarding Acute Rheumatism.*
By Dr. Henry Zeroni, of Mannheim.

This is an important paper, and its chief object is to direct attention to the fallacy of Bouillaud's views concerning the nature of acute rheumatism, and, consequently, likewise to the danger of pursuing this author's mode of treatment. Judging from an attentive perusal of Bouillaud's work, the following maxims appear to form the basis of his theory of rheumatism:

1. Acute rheumatism has its seat in the synovial membrane of the joints; it is an inflammatory disease, characterized by the same symptoms which denote other inflammations,—namely, swelling, heat, pain, redness, ulceration, and effusion.

2. In most cases it is accompanied by pericarditis or endocarditis, as proved during life by the stethoscope, and after death by dissection.

3. The fever which accompanies acute rheumatism is strictly inflammatory, and depends partly upon the cardiac affection, and partly upon inflammation of the lining membrane of the arteries.

4. That fever, which continues sometimes after the pain has left, depends upon endocarditis.

5. The fatal termination, when such ensues, depends upon complication with pericarditis, endocarditis, pleuritis, or some other disease.

6. The duration of the disease, when treated according to Bouillaud's method, is from eight to fourteen days.

7. This method consists chiefly in withdrawing blood. On the first evening, sixteen ounces are drawn from the arm; next day, two venesections, each of sixteen ounces, are made, and sixteen ounces more are taken by leeches and cupping-glasses. On the day following, another venesection of sixteen ounces is made, and sixteen ounces additional are taken by leeches; and, should the pain still continue, another venesection is made on the fifth day, which is generally sufficient to stop the progress of the disease.

8. If twelve pounds of blood have been taken at short intervals, enough has been done to cure acute rheumatism in from ten to fourteen days.

These views Dr. Zeroni considers as erroneous, and the practice founded upon them as most detrimental to the patient. After quoting the opinions of Galen, Sydenham, and Stoll upon the nature and treatment of acute rheumatism, he proceeds to give the following cases, as illustrative of the evil effects of Bouillaud's method.

Case i. A young man, between twenty and thirty years of age, was admitted into the hospital, on the 19th March, 1836, on account of a moderate rheumatic fever, unaccompanied by any local affection. Some gastric symptoms being present, he was ordered an emetic, and small doses of ammonia and tartrate of antimony were prescribed. Notwithstanding these remedies, the joints of the extremities became affected; but the accompanying inflammatory swelling was by no means violent. On the 24th, he complained of oppression over the whole chest, which continued to increase in spite of the application of twelve leeches and a blister, and continued doses of tartar emetic: the pulse was quick and hard.—Some improvement was obtained on the morning of the 25th, by a bleeding of sixteen ounces and the exhibition of nitre; but, towards evening, the state of the patient became much worse: the pulse was again hard and frequent, the oppression was more concentrated upon the left side of the chest, and was attended by burning, shooting pains, which prevented the patient from assuming the horizontal position, and caused him the utmost anxiety. The local articular inflammations had increased, and the heart beat violently. V.S. of thirty-two ounces, two grains of calomel every two hours, and mustard poultices to the calves, were followed by abatement of the symptoms. The anxiety and restlessness, however, soon returned; and, between the 25th and 29th, six pounds of blood were drawn at different times, fifty leeches were applied to the præcordial region, a seton was passed in the space between the fifth and sixth rib, and calomel, digitalis, opium, and ipecacuanha administered internally. The symptoms continued to get worse, and the patient died on the 12th of April. Dissection shewed inflammation and thickening of the pericardium, and effusion of serum into its sac.

Case ii. A man, aged twenty-eight, predisposed to rheumatic attacks, but otherwise healthy, was seized with rheumatic fever, in consequence of exposure to cold and wet. He was put upon low diet, and mild diaphoretics were ordered. On the fourth day, the joints became affected, and leeches were applied to them; the diseased joints were afterwards wrapped in oilskin, and a grain of tartar emetic ordered every hour, with warm lemonade *ad libitum*. On the following day, the patient made no complaints of the previously affected joints; but some of the other articulations had been attacked, and were so much swollen as to impede motion. They were wrapped in oilskin, and the patient was largely bled. The improvement was striking: the pain was removed from all the joints, except the knees, to which twenty leeches were applied. At night the patient was quiet and composed; but, after two hours' sleep, he suddenly awoke with great anxiety, dyspnœa, pain in the præcordial region, palpitations, and irregular pulse. This state continued till morning: percussion gave a dull sound over the præcordial region, and the respiratory murmur was absent. The articulations were free from pain, the skin was moist, thirst not excessive, bowels regular, urine high coloured. The patient was again bled, two grains of calomel were ordered every two hours, and mustard poultices were applied to the wrists and ankles. These measures were followed by some relief, but towards night the symptoms again became worse, and the application of eighteen leeches and a large blister to the chest brought no relief. Next morning, the pain in the præcordial region was excessive; the dyspnœa was greater, the pulse irregular. Another venesection, and other measures, had no beneficial result, and the patient died on the ninth day. Dissection shewed the pericardium filled with a troubled reddish fluid, and its inner surface covered with false membranes.

Case iii. A woman, aged forty, who had previously suffered from rheumatism, was attacked with the acute form of the disease. Notwithstanding repeated general bleeding, the articular affections changed their locality five times in six days; and all the joints, both of the upper and lower extremities, became in their turn the seat of acute pain, and swelling to such an extent as to prevent motion. On the seventh day, there was great diminution of all the symptoms, and the patient, for the first time since the commencement of the disease, was enabled to get some sleep. On awakening, she complained of restlessness and dyspnœa, and could not remain in the horizontal position. She soon began to complain of an acute pain in the præcordial region, which at times was so intense as to force her to cry out; there was dulness on percussion over that region, and no respiratory murmur was to be heard. The dyspnœa increased, and the beat of the heart became unequal and irregular. (V.S.; mustard poultices to the wrists and ankles; calomel and tartar emetic alternately.) These measures had no salutary effect; the patient went on from bad to worse, and died on the ninth day. On dissection, effusion was found in the pericardium, which was coated with effused lymph.

Case iv. A young robust woman, aged twenty-two, was suddenly attacked with acute shooting pains, which extended down the back from the shoulders to the sacrum. The pain was so excessive as to forbid the slightest touch, and the fever was considerable. Before her admission on the 14th, she had been bled without relief: indeed, the pains seemed rather to become more intense, but there were no symptoms of myelitis. Venesection was twice repeated, leeches were applied, and tartar emetic, with extract of aconite, prescribed internally. Dyspnœa appeared, and the patient died on the 17th. On dissection, the dorsal muscles were found of a deep brownish-red colour, filled with blood, but otherwise normal; the dura mater of the lumbar portion of the spinal marrow had lost its transparency, and was somewhat injected, as was the whole of the spinal dura mater. The spinal marrow itself, the brain, pericardium, and pleura were healthy; the lungs were not over-filled with blood, but their tissue contained a yellowish, bloody, ichorous fluid.

Here then, says Dr. Zeroni, are the results obtained by Bouillaud's practice. It seems, according to this physician, that art must do everything, and that the powers of nature are to go for nothing; that the disease is always advancing towards a fatal termination, which must undoubtedly ensue, unless art strikes boldly in to arrest it. Pathological anatomy, and the opinion of the many, have decided on the inflammatory nature of acute rheumatism; but will experience, demands Dr. Zeroni, confirm this opinion? It must be remembered that these and the following remarks apply exclusively to that form of rheumatism which is characterized by wandering affections of the joints, accompanied by fever; those forms in which the affection remains stationary in one joint, with or without fever, or which are marked by old chronic pains, are excluded entirely.

Acute rheumatism tends to pursue a regular course, as regular almost as that of the eruptive fevers, and is thus shortly described by Dr. Zeroni:—A patient, after suffering for one, or two, or more days from fever, begins to complain of painful swelling of one or more joints, often to such an extent as to impede the motion of the affected limbs. The tongue is sometimes thickly coated, sometimes it is as white as chalk; there is great thirst and complete anorexia; the bowels are rather slow, the urine small in quantity, and troubled; the skin is moist, the pulse quick and soft. By and by other joints are attacked, generally with relief to those which had suffered first, but sometimes the disease extends to almost all the joints of the body, producing great distress and a state of mental excitement, which often passes into delirium, especially at night; which becomes insupportable, from want of sleep, pain, and profuse perspiration. At this period the chest is liable to be attacked; the patient complains of pain, especially in the cartilages of the ribs; there is excessive dyspnœa, accompanied by a short dry cough, and sometimes palpitations. These symptoms may continue for three, four, or six days, during which the articular pains are less violent; these now again become worse, and gradually the thoracic symptoms and fever decrease. Gradually the pains in the joints disappear, the fever goes off completely, the appetite returns, and convalescence is established.

Such is the disease when left to pursue its natural course, unaffected by blood-letting, purging, narcotics, or diaphoretics; and the patient is generally reestablished in four or five weeks. But, when recourse is had to heroic remedies, if the patient escape the fate amply illustrated by the preceding cases, the disease is apt to fix itself upon one joint, and the patient becomes a martyr to the most agonizing pains, whilst the articulation runs through all the degrees of disorganization. In consequence, likewise, of neglect or unnecessary intermeddling, effusion into some of the principal cavities, or disorganization of the thoracic or abdominal viscera, may supervene; or the disease may become chronic, and last for months or years, producing in some cases a state of body difficult to be distinguished from hectic fever.

We shall now shortly give one or two cases as illustrative of the powers of nature in the cure of the disease.

Case i. A strong robust man, who had already, at a former period, suffered from rheumatism, was again attacked in consequence of exposure to cold and wet. The articulations of the right arm and foot were affected; there was considerable fever, the tongue was slightly coated, and the bowels slow. This state continued with little change till the fifth day; the swellings in particular joints increasing, or diminishing, or occasionally shifting altogether. At night, after the pains in the articulations had somewhat diminished, the patient was suddenly attacked with acute pain in the chest and great dyspnœa, accompanied by a short, dry cough. The pulse was quick, hard, and contracted; the skin moist, the urine small in quantity. This state continued three days; the articulations then became more painful, and the thoracic symptoms were relieved. The swelling of the joints gradually disappeared, and in four weeks the patient was in the enjoyment of his previous good health.

Case ii. A strong robust woman, aged twenty-five, who had formerly had a rheumatic attack, was exposed to cold and wet, and on the day following was attacked with fever, pains in the neck and right side of chest, oppression in the chest, incapability of drawing a full breath, and short respiration. (Spirit. Mindereri ℥ss. in Decoct. Alth. ℥vi.) Next day the application of a mustard poultice removed the pains from the neck, but it became more violent in the chest. The pain in the articulations of the right clavicle was almost insupportable, and the shooting pains in the chest so severe as to force the patient to cry out. Great dyspnœa and anxiety. (V.S. of four ounces, and nitre.) The bleeding produced relief, but an exacerbation soon followed. (A blister was applied.) Next day, the pains in the chest were most violent; great dyspnœa, anxiety, and debility, profuse perspirations, quick contracted pulse. (Poultices to the chest; Decoct. Alth. c. Aqua Laurocerasi.) No sleep. On the day following, the pains were less violent; the menses appeared fourteen days before the regular period, and slow but steady improvement followed.

These cases will suffice to shew Dr. Zeroni's practice: he gives other three cases, but our limits prevent us from noticing them. It appears, from the two we have quoted, that the alarming symptoms which seize the chest yield as readily, if not more so, to expectant medicine, as to the heroic practice of Bouillaud. In the second case, Dr. Zeroni was induced, by the alarming nature of the symptoms, against his better judgment, to order a small bleeding; but, in spite of the momentary improvement which succeeded, he was not long in perceiving his error, and was firm in resisting all inducement to repeat it. He regards bleeding in this disease as most hazardous practice, and affirms that most of the accidents which occur in its progress are owing far more to improper treatment than to the malignity of the disease, which, had it been left to itself, would have pursued a regular course, and ended happily. The swelling of the joints bears the same relation to rheumatic fever which the eruption of small-pox bears to its fever, and it is as absurd to set about removing the swelling in the one case by heroic remedies as it would be to try and remove the eruption in the other case. Rheumatic pericarditis is, in general, he says, an artificial disease, and furnishes the anatomical school with a remarkable instance of one of its most violent and most dreaded inflammations being the consequence of powerful antiphlogistic remedies.

[Without expressing our adhesion to Dr. Zeroni's views, we certainly regard them as worthy of attentive consideration. It is, however, but justice to M. Bouillaud to observe, that the active depletory treatment was too long delayed in Dr. Z.'s cases; while we think that, if Dr. Z. had availed himself of calomel, opium, and colchicum, as administered in this country in such cases, the results would have been less lamentable in some of the first series of cases.]

*Heidelberg Medicinische Annalen.* Dritter Band. Erstes Heft. 1837.

---

### *On the Cause, Seat, and Nature of the Asiatic Cholera.*
By Dr. Bellingeri, of Turin.

[Together with the late Numbers of the *Bullettino delle Scienze Mediche*, we have received an appendix of Memoirs on the Cholera, that has now for more than two years been raging in Italy. After a careful perusal of these papers, we cannot find that the transalpine physicians have added much to our knowledge of the pathology of the disease, and certainly they have done nothing to improve its treatment. The first and most original article is by Bellingeri, of Turin, of which we have given an analysis below. The second is an ingenious attempt, by Dr. Pucinotti, of Florence, to prove that cholera is a contagious eruptive disease; which idea he was induced to entertain from observing that there were very few persons who did not, during the period of reaction, exhibit a peculiar eruption upon those parts of the body and limbs that had been most livid during the cold stage. The next paper is upon the cholera as it appeared in Florence, in 1835, by Dr. Petri. This physician stands almost alone in Italy as an anti-contagionist, although he believes in the infectious nature of the disease that made its first appearance in Florence immediately after the arrival of a ship from Marseilles, (at that time an infected port,) with two of the crew labouring under the disease. The persons most affected were the inmates of the Lunatic Asylum, of whom it destroyed about fifty, notwithstanding that every precaution was taken to prevent communication between the healthy and diseased. The fourth article is a Report of the Medical Committee of Turin, published by order of the Board of Health. Bellingeri was a member of this committee, and appears to have had a considerable share in the labour of drawing up the report; which opinion we have formed, not only from the similarity of style and diction in the two documents, but because that portion of the report which is devoted to the pathology of the cholera is quite in accordance with the views given by him in his own memoir, where, however, they are more fully developed, and made to apply to every form and variety of the disease.]

The occasional (exciting) cause of cholera is a principle that, like other contagions, is communicable from one individual to another, by immediate or mediate contact; inasmuch as it adheres to solid porous bodies, that serve as its vehicles; and it is also volatile, and diffusible in the atmosphere to sensible distances. The most conclusive proof of this diffusibility is the sour yeasty smell and the harsh metallic taste observed where many cholera patients are collected together, indicating the altered state of the cutaneous and pulmonary exhalations, to which the contagious quality adheres, and is thus transmitted with ease, and as it were by preference, through the medium of the respiratory organs. The miasm of cholera, however, requires for its development and epidemic propagation a certain morbid constitution of the atmosphere, which, although incapable of generating the contagious principle, favours its extension when produced, and induces a state in the human economy adapted to its reception. Hence, the most appropriate name for the disease is the *Pestilential Cholera*, as distinguishing it from the sporadic form, as well as indicating its mode of propagation. It was the immoderate rains, and consequent unwholesome state of their food, particularly of the rice, that, debilitating the natives on the banks of the Ganges in 1817, superadded a contagious tendency to the cholera and other bilious diseases previously endemic in those regions. Like other contagious diseases, the cholera requires a certain predisposition in the individual for its reception, that experience has shewn not to be very common in Europe, even in those places where it has raged with the greatest violence.

The poison of cholera differs from all other miasmata in having a tendency, like prussic acid, to exhaust and destroy the vital principle, while the others act by irritating and stimulating it. At the invasion of the disease, the nervous system is the part primitively affected, and of this more especially the ganglionic portion, either in toto or in its principal divisions in the neck, chest, and abdomen. When the abdominal portion is affected, the disease is either confined to the digestive tube, constituting *cholerine*, or it radiates from the solar plexus to the thoracic and cervical divisions, and thence to the cerebro-spinal system, exhibiting itself in its most complete form. If the disease first lights upon the cervico-thoracic part of the ganglionic system, instantaneous death, or "ganglionic apoplexy," may result from the sudden exhaustion; or a more prolonged form of collapse, almost equally fatal, but unattended with vomiting or purging, and hence termed "dry cholera," may be induced. This, again, may transmit its influence to the abdominal ganglia, and by sympathy to the cerebro-spinal system; and thus at last the perfect form of the disease becomes established. The primary affection of the cerebro-spinal system is clearly indicated by headach, vertigo, &c., followed by loss of muscular power. The complaint, however, never stops here, but passes on, through the intercostal nerve, to every part of the body, completing the circle of diseased actions.

The most plausible explanation of these various forms of disease is to be sought in the different constitutions of the individuals, or in the mode by which the contagious miasm has penetrated the system. If it has been introduced by the saliva that has been swallowed, gastro-enteric symptoms will arise, in the form of cholerine at first, afterwards passing into the more severe species, according as the morbid matter extends its influence; if by the air respired, instant death, or "dry cholera," will arise, from the mass of the blood being poisoned at its source, and infecting every organ as it pervades it through the arteries; whereas, when the contagion insinuates itself through the skin or digestive organs, it is the veins and lymphatics that convey it, diluted and modified by their contents, back to the heart, to be still further modified and purified by the act of respiration; lastly, if the morbific matter be applied to the skin, either the digestive organs are first affected, through their sympathy with the skin, or else the cerebro-spinal system, particularly if much terror or depression of spirits be present.

The cholera is essentially and originally an affection of the ganglionic system or intercostal nerve, radiating to the heart and the whole of the sanguiferous organs, to the lungs, the digestive tube, and to the cerebro-spinal axis; and, from the debilitating nature of this affection, it may be denominated a "ganglionic paralysis."

In general, the course of the symptoms exhibits disturbance of the system in the invasion, of prostration in the cold stage, and of excitement with a tendency to inflammation during reaction. It is not true Asiatic cholera unless there is an affection of the heart and vascular system, which, in the fulminant cholera (*il colera fulminante,*) with instant death, is a real paralysis of the heart. The affection of the lungs in the cold stage arises from the diminished supply of nervous influence from the intercostal nerve, by which cause the vital turgor of the lungs is destroyed, and the circulation through them is obstructed; as is proved by their state of flaccidity and congestion with black blood after death, although this latter state is partly owing to the want of power in the heart itself. The absence of the chemical action of the lungs upon the respired air, and the cause of the blueness of the skin, must be sought in the diminished or deficient influence of the pneumogastric nerve upon the lungs.

The morbid affection of the stomach and bowels is not necessary to constitute cholera; consequently, the essence of the disease cannot be seated in them. The cholerine, however, is limited to these organs, beyond which it does not extend; either because, in this form of disease, there is not sufficient morbific matter to affect the whole body, or that, through an idiosyncrasy of the individual, it is not absorbed: indeed, the vomiting and diarrhœa may be considered as critical discharges of a salutary nature, serving to eliminate the material cause of the complaint. Even the discharges in real cholera, when not excessive, may have the same result. During the cold stage, the digestive tube is in a state of irritation, with a tendency to inflammation, as indicated by the burning at the stomach and desire for cold drinks, &c., and confirmed by dissection, that exhibits the stomach and bowels congested with black blood, which lays the foundation for a real inflammation during the reaction. The typhoid period follows the reaction, or immediately succeeds the cold stage, and is produced by the congestion in the arteries, as well as in the veins of the head, of the black blood that has not yet been thoroughly arterialized, and therefore is incapable of properly supporting the functions of the brain.

The following is Bellingeri's summary of his views:—The ganglionic paralysis of the intercostal nerve first induces a disturbance of the heart and vascular system, that may be either of an irritative or adynamic character: this causes venous congestion, chiefly of the brain and heart, that, if very great, produces death—if moderate, prepares the succeeding period of reaction, which latter, if mild, carries off the disease in perspiration, but, if severe, requires moderating by antiphlogistic measures. The affection of the intercostal, propagated to and diffused over the digestive tube, either in the invasion or cold stage, always produces irritation there, and, during the reaction, a real inflammation. The cerebro-spinal affection usually accompanies the Asiatic cholera, and is sometimes primary, sometimes secondary, and also sympathetic. In the invasion it is irritative; it is a state of collapse in the cold stage, and then of congestion, particularly in the typhoid period, the blood being thick and black.

Asiatic cholera, in its form and course, is analogous to intermittents in their different stages, especially to the fatal cold ague, (*perniciosa algida,*) combined with the choleric form of ague. The typhoid period of cholera resembles ordinary typhus fever.

[Such are Bellingeri's views, and his treatment is in accordance with them; but, except in the use of bleeding or leeches, it is inert in the extreme, being confined to warmth externally applied, and diaphoretics and aromatics in the cold stage, and diluents during reaction.]

*Appendice al Bullettino delle Scienze Mediche di Bologna.* Fasc. i. 1836.

---

### CASES OF SEVERE LESION OF THE BRAIN.

[The following cases are very interesting. They sufficiently prove how little we know of the precise functions of the encephalon, and what an extent of observation and comparison of facts are necessary before we can pronounce, with any thing like certainty, on the subject.]

1. *Case of Fatal Disorganization of the Brain, without corresponding Derangement of the Intellectual and Moral Acts.* By G. W. Boerstler, of Lancaster, Ohio.

In August, 1833, I was called to see William Miller, a lad about eleven years old: he had just received a kick from a newly shod horse, which fractured the right superior portion of the os frontis, and the anterior portion of the right parietal bone. During the operation of removing the fractured bones, I found one portion, an inch and a half long, of an irregular triangular form, driven into the right anterior lobe of the cerebrum, to the depth of an inch: on removing it, about a table-spoonful of brain was discharged. The piece of bone, having its edges serrated, and being driven from before backwards, necessarily produced a very great laceration of the meninges. The common integuments over the fracture were much contused and lacerated, and sloughed in the course of a few days, leaving exposed a very considerable portion of the skull and brain. I moulded to the convexity of the cranium wet pasteboards, and then saturated them with albumen, which, when dry, gave them considerable firmness: these I confined with the double-headed roller. I looked upon these precautionary measures as important; for I feared hernia cerebri: four days gave reality to those fears; hernia came on, but, after six days' perseverance, I succeeded in preventing any further protrusion. There was no compression, save by the fractured pieces, which were readily removed. The boy's faculties were not destroyed, but there was some intellectual confusion, from the time of the injury, during the operation, and for two hours after; from which time he recovered every faculty of the mind, and they continued vigorous for six weeks, and to within one hour of his death, which took place on the forty-third day. During all this period there was little apparent derangement in any of the organs, except a slight irritative fever, which supervened sixteen days after the injury, and continued to the termination of the case. So slight was this fever, that, in despite of all entreaties, the patient sat up every day, and frequently walked to the window and withdrew the curtain, in order to see the boys play in the streets, in which he took deep interest, frequently laughing at their gambols. Four hours after death I proceeded to the examination, in the presence of Drs. Edwards, Ohr, and Newcomer. Upon removing the cranium, the dura mater presented strong marks of inflammation over the entire arch of the head, being deeply injected in parts, and having depositions of coagulable lymph in others. From the antero-inferior angle of the right parietal bone, in a line back to its junction with the occipital, the dura mater was disorganized in three points by ulceration. The space of the skull, previously occupied by the right anterior and middle lobes of the cerebrum, presented a perfect cavity, the hollow of which was filled with some sero-purulent matter, the lobes having been destroyed by suppuration: the third lobe was much disorganized. The left hemisphere was in a state of ramollissement down to the corpus callosum. It was so much softened that the slightest touch would remove portions; and, with the aid of a sponge, I wiped away its substance to near the corpus callosum, when it began to be firmer, but presented more the appearance of a homogeneous mass than of regular organization. The chiasm of the optic nerves, as well as their entire tract, was so soft as to yield to a slight touch with the handle of the scalpel, and the olfactory were in the same condition. The corpus callosum, thalami nervorum opticorum, and tubercula quadrigemina presented no pathological condition. The cerebellum and medulla oblongata were in a physiological state. The spinal column was not examined. This boy was remarkably intelligent. In my daily visits I held frequent conversations with him, and in all my observations I could not discover the slightest derangement of his intellectual faculties; no dulness of sensibility, no obtuseness of perception, no impairment of judgment, no want of memory, and, so far as mind is concerned, he gave no evidence of disease. His vision, audition, and voice were unimpaired.

We have here a case which presents that portion of the brain from which the nerves arise, in a physiological condition, and the general nervous apparatus in a sound state, fit for conveying impressions; whilst the organ upon which depends perception and the perfection of ideas is in a great degree lost, and what remains is in a highly pathological condition; yet we have all the manifestations of intellect, as if the encephalon were not required in those highest functions. His case contradicts the opinion of Sir Charles Bell, that disease of the general surface of the brain is always attended with derangement of the mind; and it is equally opposed to the views of Desmoulins, Gall, Spurzheim, and others, who contend that the seat of intellection is in the periphery of the brain or its convolutions. In like manner, the opinion of Magendie is contradicted, that the sense of sight is always destroyed by removal of the cerebral hemispheres; for here the right hemisphere was destroyed, and yet vision was perfect with either eye. Where, I would ask, were the functions of mind executed in this case? Intellection was performed,—

the moral faculties were exercised,—and that portion of the brain in which we believe those important and complicated actions are generated and perfected was either gone or in a highly pathological state.

2. *Case of Abscess of the Cerebellum, communicated to the Académie de Médecine, the 27th Sept.* 1836. *By* M. Bouvier.

A man, fifteen years of age, had been subject for a length of time to a discharge from the ear, with deafness and frequent headach. He was suddenly seized with more severe headach in the left side of the head, vomiting, and disorder of mind. His symptoms were indeed so characteristic that a physician, who was consulted, pronounced him to be labouring under abscess in the head, and that death was almost certain.

He entered the Hôtel Dieu on the 15th of September, three weeks after the last exacerbation, when he complained of fixed pain in the head, which frequently caused him to cry out; sensibility in other respects obtuse; slow answers; somnolency; face pale, features sunken; look sad and anxious; a copious purulent discharge from the left ear; deafness of the same side; pulse slightly slower; vomiting; constipation; the movements of the limbs were preserved; an incomplete paralysis of the upper eyelid being alone observed.

These symptoms continued for the following days without any marked aggravation; and it seemed probable that the patient's life might still be prolonged for some time, when, on the 23d of September, after vomiting, accompanied by great agitation and violent outcry, he suddenly fell into a state of complete collapse. Respiration became embarrassed, and he died eight days after his entrance into the hospital, with symptoms of asphyxia.

On examining the body, there was found, as had been foretold during life, a caries of the petrous portion of the temporal bone, and an abscess in the interior of the cranium. But, what was remarkable, the abscess occupied the left hemisphere of the cerebellum, although nothing led to the suspicion that there was any lesion of that organ. There was an extensive cavity, which invaded the two outer thirds of the left lobe of the cerebellum, and which contained several table-spoonfuls of pus, somewhat similar to that of an abscess. The substance forming its parietes was softened, and of a livid tint. The meatus auditorius was filled with reddish vegetations.

The caries occupied the base of the pars petrosa only; the labyrinth and auditory nerve were untouched. There was no perceptible communication between the internal abscess and the abscess of the caries. The disease of the bone, however, extended to the dura mater, in two very circumscribed points, at the upper and hind part of the pars petrosa. The dura mater opposite these points was deeply coloured; and the coloration extended to its inner surface, where it was in contact with the cerebellum.

The cerebral ventricles were, moreover, greatly distended by a limpid fluid, and the pia mater exhibited a decided injection under the anterior part of the cerebral lobes, chiefly on the left side.

"Two circumstances," says M. Bouvier, "give interest to this case. The first is the almost entire separation, by means of the dura mater, (which was scarcely affected,) between two lesions, one of which must have been the effect of the other; so that it is difficult to explain, merely by continuity of tissue, the transmission of the affection from the ear to the cerebellum.

"The second is the absence of all the symptoms which have been of late regarded as an effect of lesions of the cerebellum; such as augmentation of the general sensibility, loss of equilibrium, and excitation of the genital organs. Could this peculiarity be owing to the slow development of the affection, or to its not having extended sufficiently far from the side of the medulla oblongata?"

*American Medical Intelligencer. April* 1, 1837.

*On the Efficacy of Sugar of Lead in Cases of Fever with Ulcers of the Intestines.* By Professor NASSE, of Bonn.

[THE treatment recommended in the following article has recently acquired a fresh interest from the recommendation of the same remedy in the Asiatic cholera.]

A fever has prevailed for some time at Bonn, attended by a peculiar appearance of the tongue; pain in the abdomen, especially on the right side, upon pressure; nervous symptoms, and a particular state of the pulse: it is also accompanied by diarrhœa, and, on examining those who had died of it, the termination of the small and the commencement of the large intestines have been found in a degenerated state. During the inflammatory stage of the disease, Dr. Nasse applied cupping-glasses to the abdomen, and in most cases more than once; in a few cases, when the pulse was hard, he bled from the arm; and in every case applied blisters to the abdomen. As soon as the diarrhœa made its appearance, sugar of lead was prescribed, generally without being combined with opium, and was continued until the fæces reassumed their natural appearance, and until the abdomen ceased to be pained by pressure. Dr. L. Jung, in his work *De Dothienenteritide ejusque plumbo acetico sanandi ratione,* had already described the admirable operation of this medicine. Dr. Nasse gives a similar testimony to its value, and states that, in his own practice, it has, in his opinion, saved several lives, which would have been lost without it. Nineteen cases of the above fever were treated with sugar of lead; five in children, and fourteen in adults. Of these eighteen recovered. The lead was not administered till the fifth, eighth, or tenth day of the complaint, or even later, when cerebral congestion rendered necessary the application of leeches. The dose was one-fourth, one-third, and half a grain, from three to six times daily. Generally, two grains were sufficient to put a stop to the diarrhœa; but, in five cases, four grains were necessary. One of the patients, who had forty stools in twenty-four hours, and who suffered from tenesmus, took in all eight grains, at first without, and afterwards combined with opium, and at last with starch clysters. The young man, who died, took altogether ten grains: in his case, the diarrhœa yielded to it in a great degree, but the abdominal irritability and the tympanites were such as to induce its fatal termination. As soon as further doses of lead seemed unnecessary, a weak infusion of ipecacuanha was given. In some of the cases which recovered there had been petechiæ; and in some, too, the evacuations had been bloody; but the lead had been administered all the same. Opium was not generally ordered, on account of its effect upon the head. In eight cases, on account of weakness and fainting fits, the liq. ammon. carb. was had recourse to; in one, that of a child, quinine was given before the lead, but, the fever increasing, and blood passing off with the stools, the latter was prescribed, and with the best effect. *Med. Zeitung v. Ver. für Heilkunde in Pr.* 1836. No. 23.

---

*Moral Management of the Insane in America.*

[THE following extracts from the Report of the Maclean Asylum, in Boston, for the year 1836, place in a striking light the benefits to be derived from a rational treatment of insanity. Both the treatment and the results correspond, in a striking manner, with those recorded in Dr. Macrobin's recent Report of the Aberdeen Asylum.]

The number that enjoyed the advantages of the institution during the year was one hundred and eighty-three, of whom one hundred and twelve were discharged on the 1st of January last. Of these, sixty-four had recovered; seven were convalescent: two much improved; five improved; nine not improved; fifteen were sent away by order of committee; ten died. Of the nine discharged, "not improved," five were hopeless cases of masturbation at the time of their admission; two were idiotic, and two had insufficient trial. Of the deaths, one was of phthisis pulmonalis; one of old age; one of mania with convulsions; one of dysentery; one of suicide; two of marasmus; one of fracture of the neck of the thigh bone; and two of acute inflammation of the mucous membrane of the bowels.

"Our amusements are various and numerous. We keep a carriage, two carioles, one chaise and four horses, which are devoted almost exclusively to the use of the patients. Many of them ride every fair day, and have, the last year, ridden ten thousand miles. The males are also engaged at bowls, quoits, bass-ball, fishing, fancy painting, walking, dancing, reading, swinging, and throwing the ring, &c. Of the one hundred and three male patients who have been in the institution during the year, seventy have been engaged in out-door amusements, passing, in this way, three thousand five hundred and forty-one hours. Seventy-seven have walked ten thousand four hundred and thirty-one miles. Some have walked, individually, over one hundred and fifty miles per month. Twenty-four have occupied one hundred and nineteen hours in fishing.

"In our 'Labour Department' the patients have been equally active and interested. Seventy-seven of the males have engaged in manual labour, and have worked, allowing six hours per day, (more than which no patient has been asked to work,) one thousand seven hundred and ninety-eight days.

"Gardening, the cultivation of flowers, and farming, as usual, have occupied and interested many of the patients during the whole season. The tastes and wishes of each individual have been, in all cases, consulted as far as possible; and while some were engaged with the team, others would be equally ambitious to excel in planting, hoeing, or in displaying their taste in the arrangement of the flower-beds and borders. Thus their irritability was expended in healthy exercise and occupation, and instead of meeting them in the halls in tattered garments with oaths and imprecations, we are greeted in the walks with the affectionate grasp of friends, their countenances glowing with pleasure and contentment, and each commenting, in his own way, upon the business of the day.

"Nor has our labour resulted in mere amusement, as the harvest of our crops abundantly testifies. Our farm and lands, inclusive of all the grounds occupied by the buildings and courts, consist of twenty-five acres. We have raised, for the most part, vegetables enough of every kind to supply the institution for the year, and have cut hay sufficient to keep five horses and six cows, besides storing eighty barrels of apples and fifty bushels of pears. We have also made rose-water enough for medicinal and culinary purposes, and disposed of fifteen dollars' worth. The net profits of our farm and garden, for the past year, have been 500 dollars.

"In April we opened the dome of the male wing as a carpenter's shop for the patients, having previously secured the services of a judicious carpenter to superintend and work with them; and, although we were confident of success, our hopes have been more than realized. Not the least accident has occurred, although the patient's have not been restricted in the use of tools, and herein, as I conceive, our safety lies. The patients feeling themselves under no restriction, consider that they are placed upon their honour, and their self-respect being called into action, they would not forfeit the confidence and good opinion of the officers for any consideration. Give a man constant employment, treat him with uniform kindness and respect, and, however insane he may be, very little need be feared from him, either of mischief or violence.

"Fifty patients have worked in the shop, at six hours per day, and have been employed eleven hundred and fifty-one days; and made seven thousand two hundred and thirty-six boxes, which have been sold for 907.06 dollars.

"In cases of masturbation, we depend entirely on labour for restoration. During a residence of ten years in this asylum, I have never known a single case of masturbation to be cured, unless the patient engaged in regular labour. This is a very large and most unfortunate class of the insane. We seldom receive a case of this kind in its incipient stage. It is so insidious in its approach, that before the friends of the patient are aware of his situation, he is past recovery. Labour promises the only relief. More improvement in this class has been evinced the past year, than in all the others together, and work alone has effected it.

"The results in the female wing have been equally interesting. Fifty patients have been received. Of this number have recovered, 30. Convalescent, 8. Much improved, 5. Improved, 3. Died, 4. Total, 50.

"The Belknap Sewing Society continues its operation, and affords agreeable occupation and diversion for its members. They continue their regular weekly meetings, which are held in the oval room of the mansion-house, or in one of the halls of the wing. In the absence of the presiding officer, the meeting is organized by choosing, on nomination, by a vote of a majority, one of the members to act as president *pro tem.*, whose duty it is to oversee the work and read some interesting story selected for the occasion. Their employment is piecing and quilting bed-coverings, and making and mending garments and furniture for the Institution and the patients. After the labours of the day are over, tea is passed round, and then the meeting adjourns. The account of each day's proceedings is recorded in the Society's book. It is sixteen months since the Society was organized, and the avails of their work have been, in cash, 112.96 dollars.

"In all our amusements and recreations, it is our intention to blend utility with labour or diversion. Thus, when we walk or ride, some object of interest is sought to visit; and in this respect the advantages of the locality of the institution are pre-eminent. It stands in the midst of the most interesting portion of New England, isolated from the noise and throng of business, but in full view of the capital and its beautiful environs. In these excursions the patients have uniformly conducted themselves with perfect propriety.

"Following out this plan, (of the combination of labour with utility and pleasure,) the Belknap Sewing Society is professedly and operatively benevolent. They furnish clothing for any of their members who may be needy, and sometimes purchase for themselves articles of taste and fancy; and they seek out and assist the afflicted and destitute of the neighbourhood. The poor widow, whose husband was killed in a sudden and shocking manner, last summer, by the railroad engine, was visited, and mourning was provided for herself and daughter at the expense of the Society. They called a special meeting, and deputed a member to purchase the articles necessary; and, with their accustomed promptness, made them with their own hands. I mention this, not as an act of charity worth naming, but as exemplifying the fruits of a system of moral management which is pursued, and to show that our patients are not excluded from society, and that there is scope enough for useful occupation even here. The making of the dresses for this widow and her daughter, for the time, engaged the united interest and attention of all. Diseased manifestations were quieted in the universal feeling of sympathy for that afflicted family. This being over, something else would be found to excite a similar interest, and a succession of objects, to engage their attention and to call into exercise the better feelings of their nature, has helped to do away, little by little, diseased impressions, and bring about, with many, the healthy and natural operations of the mind and body.

"Our social meetings, for diversion and recreation, continue to exert a benign influence on the convalescent. The weekly dances are continued with unabated interest, and the deportment of the patients, without a single exception, has been respectful and appropriate. Fifty-four of the males and fifty-two of the females have attended on these occasions. A sure guarantee against all improprieties is found in the constant attendance of both nurses and officers, who take an active part in the amusements. We assemble at an early hour of the evening, and the recreations consist in alternate dances and marches, with occasional songs, accompanied by the piano. At eight o'clock refreshments are served, and at nine the party 'breaks up.' For two or three days before the party, the females are engaged in preparing their dresses for the occasion, and for some days after they have a fund for remark in the events of the evening. The males also are found practising the figures of the dance, and perfecting themselves in the marches, during the week. The females have, besides, meetings every afternoon during the winter season, at which they read one hour, and pass another in practising upon the piano and in the exercise of dancing.

"Our religious meetings and exercises have been continued, and with all the success which the trial of last year led us to anticipate. Seventy-nine of the males and sixty-six females have attended family prayers. Not the least disturbance has been witnessed; but a great degree of solemnity, suited to the occasion, has universally been maintained, and the patients of both departments, with a few exceptions, depend as much upon being present at this exercise as upon their daily meals. The attendance at prayers is altogether a matter of choice.

"Our females, the past year, have ridden some thousands of miles, walked in the country 1,159 miles, walked in the garden 150 hours, folded and ironed clothes 1,025 hours, and assisted in domestic concerns 1,025 hours.

"In addition to the work before stated as having been done by the males, they have sawed, split, and piled all the wood for the whole establishment, viz. 200 cords; and have carted 100 cords from the wharf to the house. Work promises much; and it has been the aim of the Institution, the past year, to keep every patient employed in labour as far as possible. One patient has braided and sewed 100 palm-leaf hats." *American Medical Intelligencer. May* 1, 1837.

---

### *On the Treatment of Delirium Tremens by Digitalis and Opium.*

I. *On the Infusion of Digitalis in Delirium Tremens.* By Dr. Magnus Huss, of Stockholm.

The infusion, prepared with a drachm of the leaves to a pint of water, was administered in six cases, all of them men of strong constitution, of from twenty-four to thirty-three years of age. Two of the cases required bloodletting. In three, a table-spoonful of the infusion was administered every hour throughout the day only; in the other, a like quantity was exhibited every hour both day and night. In the former number, the disease yielded, and sleep ensued on the third day; in the latter number, sleep ensued in thirty-six hours; an equal quantity of the infusion was thus required in both cases. The patients awoke after a sleep of from six to ten hours, free from the disease, but labouring more or less under the effects of the medicine. In one patient the pulse had sunk to thirty-five beats, but in the other the rhythm was normal; in the whole number the pupils were contracted, and they complained of dryness of the mouth, burning in the throat, humming in the ears, heaviness in the head, great weakness, and nausea; which last symptom was so severe in one patient that for two days he vomited whatever he took.

*Jahrbücher der Gesammten Med.* B. xv. H. 1.

II. *On the proper Dose of Opium in Delirium Tremens.* By Dr. Weisse.

A case of delirium tremens, in which one grain of opium, taken every hour up to fourteen doses, failed in effecting a cure, but removed the delirium. The friends became alarmed by observing that the patient squinted incessantly, saw double, and that the face became horribly distorted. These symptoms were attributed by Dr. Weisse to the opium producing too little effect; he accordingly ordered two-grain doses every two hours. After the second dose the patient slept soundly, and awoke quite well. Dr. W. observes, that when a patient labouring under delirium tremens is under the influence of opium, and his mind, instead of picturing to him men and devils, begins to busy itself with *insects*, he is about to fall asleep.

*Zeitschrift f. d. Gesammte Med.* B. v. H. 2. *Hamburg, June,* 1837.

---

### *New Mode of treating Empyema.* By M. Reybard.

M. Reybard cites three cases of empyema. In the first case the patient, sixteen years of age, fell upon the side, which produced pleurisy, and which was followed by an effusion. The second, forty years of age, received, in a state of drunkenness, a blow from a knife on the anterior and superior part of the chest, from which resulted an effusion of blood. The third, aged seventeen years, had a spontaneous pleurisy of the right side: this was followed by an effusion, which, instead of being absorbed, collected in so great abundance that the heart was displaced. These three patients were operated on, and cured: the first in fifteen or twenty days, the second in thirty or thirty-five days, the third in four months. Besides the operation, the second had to sustain the action of detersive injections into the pleural cavity. The third case was operated on twice; the first time in the intercostal space; but the effusion being reproduced, M. R. pierced a rib, and fixed a canula in it furnished with a valve. It is to this practice he attributes, in a great degree, the success he has obtained in the treatment of empyema.

This instrument consists of a tube of silver, furnished at the end with a piece of catgut, four or five inches long. That this catgut may perform the office of a valve, it should be held in warm water until the sides are softened. A plaster of diachylum, fastened near the external extremity of the tube, fixes the latter to the walls of the chest, and at the same time that it prevents the external air from penetrating into the cavity by the wound, it unites exactly the lips of the wound to the tube.

M. R. leaves the canula constantly in the wound, convinced of the double necessity of evacuating the effused fluid, and of leaving the chest open to prevent a new collection. *Bulletin de l'Académie Royale de Médecine. April,* 1837.

---

*Case of Dyscrasy, treated with Sugar.* By Professor DUNGLISON.

WHEN sugar is added to venous blood out of the body, it immediately communicates a florid hue to it, in the same manner as many salts, which are presumed to have an alterative effect when administered internally. Some change is produced by all those alterative agencies, so that when the modified blood attains the capillaries it induces a new action in them, and breaks in upon the pathological catenation that constitutes the cachexia. When the capillary functions are morbidly affected, as in chronic cutaneous eruptions or ulcerations, there are two great methods in which we may reach the disease: the one is by the application of external remedies to the diseased capillaries; the other is by changing the impression made upon them internally by the fluid that circulates within them. Sugar (like arsenic, creosote, iodine, and other alteratives,) acts in the latter way.

The success which we have met with in the removal of inveterate eruptions from the administration of sugar, in the manner to be mentioned presently, has induced us to infer that the different alterative syrups, officinal and empirical, may be mainly, if not entirely, indebted for their efficacy to the sugar they contain.

Early in last November, a lady, from a distant city, came to Philadelphia, to place herself under the care of a distinguished professional friend, who advised her to consult the editor. Four years ago, her husband, who was a dissolute character, had contracted syphilis, and communicated it to her. She had applied to several physicians, but without experiencing entire relief. Ulcerations existed on various parts of the body; she had nodes on the tibia, &c.; added to which, she had been unable to sleep, in consequence of severe pains in the bones, for the eighteen months prior to her arrival in Philadelphia. In order that full opportunity might be afforded for recovery, she took a house, had her furniture sent to her, and determined to reside during the winter in this city. She had already taken various forms of mercurials, and amongst the rest had persisted, for a length of time, in the use of the solution of corrosive sublimate. The solution was, however, directed again, so that she should take one-sixteenth part of a grain of the bichloride three times a day; and, in addition to this, she was ordered to dissolve a pound of rock candy in a pint of water, and to take a wine-glassful of this solution four times a day. Under this course, the ulcers healed; the nodes disappeared; the osteocopi ceased; her nights were passed in comfort, and at the end of five weeks she was so well, that she determined to rejoin her family, quitted her house, removed her furniture, and went away in the middle of December with feelings of perfect recovery. She was recommended, however, to persevere in the plan advised. Since then we have not heard from her.

*American Medical Intelligencer. April* 15, 1837.

*Curious Case of Absorption of Bone.* By John H. Marable, m.d., of Tennessee.

The following singular case of absorption and reproduction of bone occurred in the person of a negro, who is now about ten years old. When about three years of age, a softness or absorption of the scull occurred in several places, say five or six. These would occasionally close, and be observed in other parts; and these changes have continued until within the last twelve months: at this time the nose, with all its bones, one of the maxillary bones, and those of one eye, are entirely absorbed and soft, so that the pulsations of the brain can be as distinctly felt as those of the wrists. His intellect is somewhat impaired, but not until lately. The eyeball of the side affected is protruded, or rather appears so, from the absorption of the bone. All these appearances I have examined within the last day or two. He has a great appetite, sleeps much, and can walk about pretty well. There is no doubt that at this moment more than half the bones of his scull and face are wholly absorbed. His condition is attributable to no known cause; he is of usual size, and the rest of his body (the head being a little too large,) is of the usual form and proportions. It is a rare case, and no relief is expected; but the master of the boy, as well as myself, would like to know if such a case has ever come under the care of Dr. M'Clellan; and, further, his opinion relative to it.

*American Medical Intelligencer. April* 15, 1837.

